# Mesenchymal Stem Cells in Sepsis and Associated Organ Dysfunction: A Promising Future or Blind Alley?

**DOI:** 10.1155/2017/7304121

**Published:** 2017-09-14

**Authors:** Jan Horák, Lukáš Nalos, Vendula Martínková, Jan Beneš, Milan Štengl, Martin Matějovič

**Affiliations:** ^1^1st Medical Department, Faculty of Medicine in Pilsen, Charles University, Prague, Czech Republic; ^2^Experimental Intensive Care Unit, Biomedical Centre, Faculty of Medicine in Plzen, Charles University, Alej Svobody 1655/76, Plzen, Czech Republic; ^3^Third Department of Surgery, University Hospital Motol and First Medical School, Charles University, Prague, Czech Republic

## Abstract

Sepsis, newly defined as a life-threatening organ dysfunction caused by a dysregulated host response to infection, is the most common cause of death in ICUs and one of the principal causes of death worldwide. Although substantial progress has been made in the understanding of fundamental mechanisms of sepsis, translation of these advances into clinically effective therapies has been disappointing. Given the extreme complexity of sepsis pathogenesis, the paradigm “one disease, one drug” is obviously flawed and combinations of multiple targets that involve early immunomodulation and cellular protection are needed. In this context, the immune-reprogramming properties of cell-based therapy using mesenchymal stem cells (MSC) represent an emerging therapeutic strategy in sepsis and associated organ dysfunction. This article provides an update of the current knowledge regarding MSC in preclinical models of sepsis and sepsis-induced acute kidney injury. Recommendations for further translational research in this field are discussed.

## 1. Introduction

Stem cells may be defined as cells capable of self-renewal and at the same time endowed with the ability to differentiate practically into all types of human cells. There are basically two main groups of stem cells—the first consists of embryonic stem cells, ESC, which are located in the inner cell mass of the emerging blastocyst and which may differentiate into cells of all the three primary germ layers. The second group then consists of adult stem cells, ASC, which are present in all tissues but have limited differentiation potential. Adult stem cells include haematopoietic stem cells (HSC) located in the bone marrow and representing haematopoiesis progenitors and so-called nonhaematopoietic stem cells (NHSC) of which the so-called *mesenchymal stem cells* (MSC) are a subgroup.

MSC were first described in the 1950s by the Russian haematologist A. Friedenstein. He thus followed in the footsteps of his senior colleague A. Maximow whose pioneering work is reflected in haematopoietic stem cell transplantation that today saves thousands of lives worldwide [1]. MSC are a heterogeneous group of multipotent cells, morphologically akin to fibroblasts, that form colonies and are capable of differentiation into mesenchymal tissue (osteocytes, chondrocytes, or adipocytes) [2–4]. It should be pointed out that although the term “mesenchymal stem cells” is commonly used in current literature, it does not reflect the essence of the definition of a stem cell, that is, the ability to differentiate into all cell types. The “alternative” term “mesenchymal stromal cells” is not appropriate either, as it has not been demonstrated thus far that these cells are involved in the formation of tissue stroma. However, for the purpose of this text, we will respect the general designation MSC (mesenchymal stem cells). In this article, we performed an update review on the potential therapeutic efficacy of MSC in preclinical models of sepsis and associated organ dysfunction. Literature was sourced by conducting a search of PubMed database using phrases and synonyms for “mesenchymal stem cells,” “sepsis,” “endotoxemia,” “acute kidney injury,” “organ dysfunction,” and “cardiovascular.” The search was limited to articles published from 2012 to March 2017.

## 2. Properties and Mechanisms of Action

**Figure 1:**
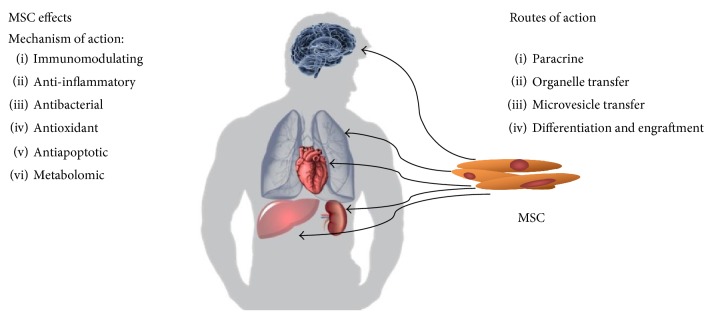
Mechanisms and means of MSC action.

MSC are currently the focus of much attention thanks to a number of their unique properties. Over the previous years, it has been demonstrated using animal models that transplantation of allogeneic or autologous MSC ameliorates symptoms caused by inflammation, ischemia, or physical damage to living tissues [5, 6]. Absence of surface MHC complex II expression enables these cells to avoid mechanisms of immune allorecognition [7] and this property in combination with the ability to suppress autoimmunity and the “graft-versus-host” reaction [8] means that MSC are a suitable basic material for potential cell therapies. Last but not least, the ability of so-called transdifferentiation has been described *in vitro*. This is a process whereby the stem cells of one germ line differentiate into cells of another germ line [2]. The reparative mechanism applied by MSC when renewing damaged tissues has not as yet been satisfactorily clarified. The concept held until recently that this involved MSC migration, engraftment, and differentiation at the site of damage appears obsolete. It has been demonstrated that during tissue repair, MSC do not migrate in a sufficient amount and do not engraft sufficiently long enough to satisfactorily explain tissue reparation via this mechanism [9]. Spees et al. have summarised the possible mechanisms of action used by MSC during the process of tissue repair [9] and these are shown in Figure 1:

1. Differentiation of MSC into the cells of the damaged tissue
2. Reparation of damaged cells by their fusion with MSC
3. Paracrine secretion of signalling molecules that stimulate tissue repair/prevent further damage has immunomodulatory functions
4. Transport of organelles and/or molecules from the MSC into the damaged cell via tunnelling nanotubes (TNT)
5. Molecule transfer via exosomes or microvesicles that separate from the MSC.

**Figure 2:**
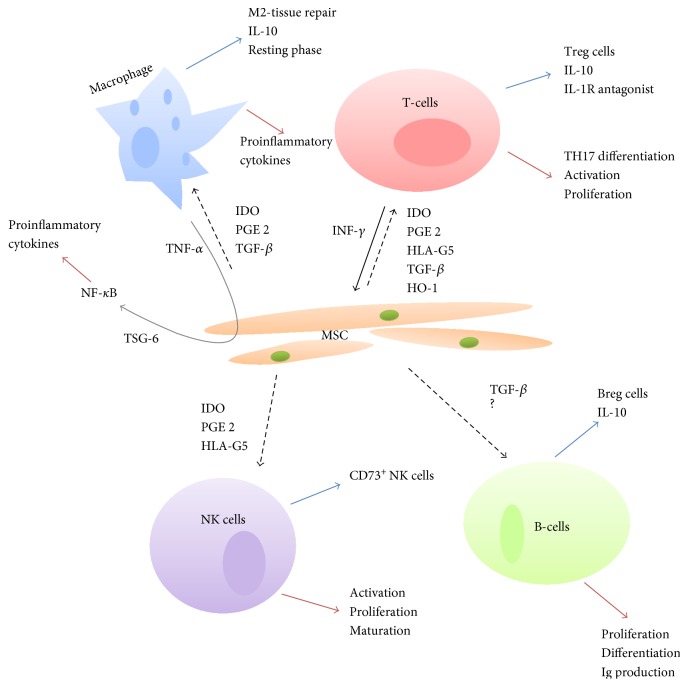
Known mechanisms of MSC immunomodulatory activity in sepsis.

The means by which MSC modify immune system processes are being studied intensively, as it is the process of immunomodulation and the possibility of influencing the course of inflammatory reactions that has made MSC the focus of great attention in experimental intensive medicine.  Figure 2 describes the pathways known to date through which MSC paracrine secretion affects immunocompetent cells.

One of the MSC molecular immunomodulatory mechanisms involves suppression of proliferation and activation of T-lymphocytes with concurrent activation of T-regulatory lymphocyte (Treg) proliferation on the basis of *IDO* (indoleamine 2,3-dioxygenase) and *prostaglandin E2* (PGE2) secretion following MSC stimulation by INF-*γ* [4, 10]. Macrophage stimulation via PGE2 and IDO leads to increased expression of anti-inflammatory IL-10 [10]; PGE2 and IDO also inhibit differentiation, maturation, and the process of antigen presentation by dendritic cells [10]. In costimulation with the MSC-produced *HLA-G5* (human leukocyte antigen G5) and IL-10, these PGE2 and IDO then inhibit activation and proliferation of NK cells and, on the contrary, potentiate the production of CD73^+^ NK cells [10–12] that play an important role in antitumour immunity [11]. Secretion of HLA-G5 then affects in a similar manner to IDO and PGE2 the proliferation of T-lymphocytes [12]. Other cytokines produced by MSC include *TGF-β* (transforming growth factor-*β*) [10, 13]. This protein, produced by all cells of the myeloid haematopoietic lineage in three isoforms (TGF-*β*1–3), ranks among polyfunctional cytokines. It stimulates Treg in the sense of IL-10 production, prevents differentiation of T-helper lymphocytes into the TH17 form producing proinflammatory cytokines, inhibits B-lymphocyte proliferation [14], and last but not least inhibits macrophage activity and by inhibiting NF-*κ*B decreases the production of proinflammatory cytokines within macrophages [13, 15]. *HO-1* (heme-oxygenase-1), also produced by MSC [10], is another cytokine with an immunomodulatory function. Expression of HO-1 by MSC is induced by proinflammatory interleukins. The role of HO-1 in the process of immunomodulation involves stimulation of Treg and production of IL-10 [10, 16] and production of the IL1R (IL-1 receptor) antagonist as well as induction of mitochondrial biogenesis [16]. The *TSG6 protein* (TNF*α*-stimulated gene protein 6) secreted by MSC following their stimulation by TNF*α* also plays a role in the inhibition of the expression of proinflammatory cytokines (via NF-*κ*B) through negative feedback [10, 17].

The protective effect of MSC on damaged cells and tissues or those exposed to stress may also be mediated by the mechanism of organelle or functional molecule transfer via so-called TNTs (tunnelling nanotubes) [9]. This mechanism was originally described in LPS-induced ARDS in mice [18] and later also in tissues outside the lung parenchyma [19] including tumour tissues [20]. These are protein channels of the gap junction type consisting of F-actin [21] and connexin 43 [18]. Active substances and some organelles, especially mitochondria, are transferred via these channels, which enable MSC to increase ATP production and thus partially or completely restore bioenergetic processes within a damaged cell.

Apart from immunomodulating properties, MSC are also endowed with the ability to directly affect the infectious agent [22]. These antimicrobial effects are mediated on the one hand by secretion of antibacterial peptides (LL-37 or lipocalin-2) and on the other by intensification of phagocytosis following MSC-induced transformation of type 1 macrophages into type 2. Devaney et al. [23] demonstrated in a mouse model of *E. coli*-induced pneumonia a lesser intensity of lung damage, lower bacterial load, higher intensity of phagocytosis, and higher levels of LL-37 following the intratracheal administration of MSC.

The antioxidant and antiapoptotic effects of stem cells also play a protective role in the process of organ damage [9, 22]. The products secreted by stem cells help ameliorate oxidative tissue damage (especially of the lungs, liver, and kidneys) [22]. Some recent works using animal models of sepsis-induced organ damage also describe MSC-associated increased secretion of a whole range of growth factors [23, 24].

## 3. MSC in Sepsis

Table 1 summarises studies dealing with the utilisation of MSC in the treatment of sepsis using preclinical models. The study of the Japanese team [25], which used the intraperitoneal application of adipose tissue-derived mesenchymal stem cells (A-MSC) in the treatment of toxic shock syndrome (TSS; sepsis induced by staphylococcal enterotoxin A) potentiated by the application of a lipopolysaccharide in a mouse model demonstrated a lower 40-hour mortality (A-MSC versus placebo—73% versus 87.5%) and suppression of INF-*γ*, TNF-*α*, IL-6, and IL-2 expression measured 18 hours after induction of sepsis. On the other hand, Kim et al. [26] did not demonstrate a significant positive effect of MSC on mortality in a mouse model of TSS induced by staphylococcal enterotoxin B. However, the authors describe a decrease in the level of proinflammatory cytokines IL-2, IL-6, and TNF-*α* in the group of mice who received MSC compared to the control group. Ou et al. [27] compared the effect of MSC and placebo on mortality, evolution of biochemical markers, and expression of pro- and anti-inflammatory cytokines during LPS-induced sepsis in a mouse model. The BMSCs and ADMSCs significantly reduced mortality rates and majority of proinflammatory cytokine levels. Their work then compared the effect of the individual conventional types of MSC (adipose-derived MSC (A-MSC) and bone marrow-derived MSC (BM-MSC)) on the course of sepsis with decreased concentration of IL-8 in the group treated with A-MSC compared to the BM-MSC group. Pedrazza et al. [28] studied the effects of A-MSC application in a mouse model of sepsis induced by the administration of *E. coli* into the peritoneal cavity. There was a significant decrease in 26-hour mortality in the group that received MSC compared to the control group. Following MSC application, these animals also presented with lower levels of proinflammatory cytokines (IL-6, MCP-1), significantly lower levels of alanine (ALT) and aspartate (AST) aminotransferase, and significantly lower apoptotic activity in spleen cells. Chao et al. [29] then compared the efficacy of MSC from other sources (BM-MSC and UC-MSC, i.e., umbilical cord-derived MSC). They demonstrated in an animal model of sepsis induced by cecal ligation and puncture (CLP) lower mortality of the animals that receive both types of MSC. The control, untreated group also showed higher levels of proinflammatory cytokines IL-6 and TNF-*α* as well as significantly lower levels of Treg lymphocytes (CD3^+^CD4^+^CD25^+^) than did the animals that received MSC. This study is one of the first to define the pathways of the immunomodulatory cellular effects of MSC in inflammation. Alcayaga-Miranda et al. [30] used MSC obtained from menstrual blood (Men-MSC) separately or in combination with antibiotics in a mouse model of CLP-induced sepsis. Their results showed the superiority of Men-MSC over those derived from bone marrow or adipose tissue (BM-MSC or A-MSC) in the inhibition of *in vitro* bacterial growth. *In vivo*, the combination of ATB (enrofloxacin) and Men-MSC represented the most effective treatment modality (i.e., reduction of 96-hour mortality). Wang et al. used an attractive source of mesenchymal stem cells (dermal stem cells (D-MSC)) in their experiment [31]. Cells acquired from the skin of newborn mice were administered to a group of animals with CLP-induced sepsis. This group of animals demonstrated a milder course of the sepsis with lower ten-day mortality compared to the control group. At the same time, there was a decrease in the level of proinflammatory cytokines (IL-1, IL-6) and an increase in the level of interleukins 4 and 5. We have already mentioned the inhibitory effect of MSC products (especially IDO, HLA-G5, and PGE2) on NK cells. Their proliferative and increased secretory activity, in particular in the initial phases of the process, appears to be a factor negatively affecting the course and mortality of septic states according to studies published so far [32–34]. In a recent study of Liu et al., the authors demonstrated inhibitory effect on proliferation and maturation (identification of CD3e^+^ forms) of septic NK cells (sNK) following their culture together with MSC *in vitro* [35]. In addition, the authors demonstrated a lower level of circulating sNK *in vivo* in CLP mice 72 hours following MSC application. The 72-hour survival interval in this experiment was longer in septic mice that received MSC (60% versus 25% in CLP mice without MSC administration, versus 90% in sham controls). *In vitro* and *in vivo* determination of cytokine levels concurred with the conclusions of other experiments, with significantly lower levels of TNF-*α*, IL-6, and INF-*γ* and higher levels of IL-10 in NK cultures with cocultivation of MSC and in the model that received MSC. An interesting paper was published by Sepúlveda et al. [36] dealing with the immunomodulatory effect of senescent MSC. In their experiment, the authors created 2 types of MSC: a senescent type cultured during concurrent *γ* radiation and a second type of MSC immortalised by the transduction of hTERT (recombinant telomerase-reverse transcriptase), whereby they attempted to simulate the activity of MSC in an ageing and young organism, respectively. *In vitro*, there was no difference in the inhibitory activity of senescent MSC on the lymphocyte population compared to the immortalised type of MSC. In the *in vivo* model of endotoxemia in mice, however, application of the senescent type of MSC did lead neither to a decrease in 24-, 48-, 72-, 96-, 120-, and 144-hour mortality nor to any effect on the level of proinflammatory cytokines compared to the immortalised and wild-type MSC. The authors considered that senescent MSC were noneffective because of the decline in their migratory potential in reaction to proinflammatory cytokines, whereby the secretory and immunomodulatory function of MSC was not significantly compromised. The possibility of influencing innate immune processes, specifically the signalling pathway of toll-like receptor 4 (TLR-4), was studied by Wu et al. [37]. Activation of TLR-4 (most often by binding to a lipopolysaccharide) leads to an increased expression of proinflammatory cytokines [38]. The results of this experiment replicate the aforementioned works in the sense of lower mortality, reduction of proinflammatory interleukin levels, and increase in anti-inflammatory interleukin levels. In the group of animals that received MSC, lower expression of mRNA coding the second messenger associated with TLR-4 (protein MyD88) was demonstrated in the liver tissue. In parallel, there was also a lower ratio of phosphorylated (activated) NF-*κ*B genes.

In summary, application of MSC in rodent animal models of sepsis is associated with lower mortality, amelioration of the course of sepsis due to inhibition of proactive elements of the immune system, and a change in the pro- and anticytokine ratio both in *vitro* and *in vivo.* No study published in literature so far has demonstrated adverse effects associated with the application of MSC in animal models of sepsis.

## 4. MSC in Sepsis-Associated Acute Kidney Injury

**Figure 3:**
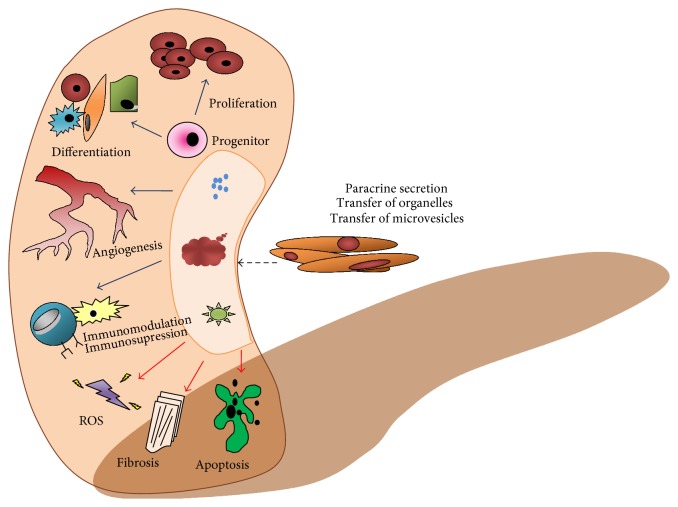
Mechanisms of the protective effect of MSC in the process of acute kidney injury (ROS = reactive oxygen species; blue arrow = process activation; red arrow = process inhibition) (adapted from [57]).

Sepsis is the most frequent cause of acute kidney injury in intensive care units (sepsis-associated AKI (S-AKI)) [39]. The pathophysiology of this process has not been elucidated satisfactorily. However, the prevailing view today is that an important role in this process is played by inflammation, together with abnormalities of renal microcirculation and changes in cell bioenergetics [40]. The mechanisms underlying the potential therapeutic effect of MSC in S-AKI are summarised in Figure 3.

Table 2 summarises studies dealing with the application of MSC in the treatment of S-AKI using preclinical models. Using a model of CLP-induced sepsis in mice that received MSC at a dose of 10^6^ cells 3 hours after sepsis induction, Luo et al. demonstrated a lower incidence of S-AKI [41]. Histological examination of the kidneys showed a significantly lower score of acute tubular damage in animals that received MSC compared to the septic controls. Another issue that this experiment addressed involved the possibility of influencing IL-7 production by MSC paracrine secretion as a potential therapeutic target within the pathophysiology of S-AKI. The research team also demonstrated significantly lower levels of all proinflammatory cytokines including IL-17 and CXC as well as CCL chemokines *in vivo* in the group that received MSC 24 hours after induction. A secondary outcome of this study involved significantly lower 7-day mortality in mice that received MSC.

The effect of MSC derived from Wharton's jelly (WJ-MSC) on the development and course of S-AKI in an animal model was studied by Cóndor et al. [42]. They randomized rats into three groups: sham, CLP-polymicrobial sepsis, and CLP + WJ-MSC (administered 6 hours from sepsis induction). This work reported lower 5-day mortality in animals from the CLP + WJ-MSC group compared to the CLP group (12.5% versus 44.4%). Insulin clearance was lower in the CLP group after 6 and 24 hours from sepsis induction compared to the control group, while the 24-hour glomerular filtration rate did not differ significantly between the WJ-MSC and control groups. Histological examination of the kidneys demonstrated greater infiltration by macrophages and monocytes in the CLP group, as well as a greater intensity of apoptosis compared to the group treated with MSC. Expression of NF-*κ*B and the levels of studied proinflammatory cytokines (IL-1, IL-6, and INF-*γ*) were also higher in the CLP group compared to the other two. However, the level of TNF-*α* and of the anti-inflammatory cytokines (IL-4 and IL-10) did not reach a level of significance in both septic groups. In their work, Sung et al. [43] focused on the effect of apoptotic MSC, that is, MSC cultured in a stress environment. In the experiment involving a CLP model of polymicrobial sepsis in mice, they demonstrated a higher efficacy of the cultured (apoptotic) MSC compared to standard cultures in terms of a decrease in TNF-*α* and serum creatinine levels after 72 hours from sepsis induction. A similar work undertaken by Tsoyi et al. [44], who used MSC preconditioned with carbon monoxide in the treatment of CLP-induced sepsis, showed not only a higher 7-day survival but also a lower incidence of AKI in a mice model. It also appears that combined therapy with ATB (ciprofloxacin) and A-MSC may be more effective than A-MSC administered alone in terms of decreasing mortality and incidence of S-AKI in a CLP model of polymicrobial sepsis [45]. In contrast, combined therapy using MSC and melatonin, which demonstrated its benefit as an antioxidant in experiments using animal models of reperfusion injury in a whole range of organs, did not prove more effective from the aspect of S-AKI incidence compared to the application of A-MSC alone in a CLP model of sepsis. However, this combination did have a positive effect on mortality, the inhibition of proinflammatory cytokines, and NF-*κ*B expression in the kidneys compared to the group treated with A-MSC only [46].

## 5. MSC and Cardiovascular System in Sepsis

Hyperdynamic circulation and myocardial depression develop in most patients with septic shock [47]. However, only a few studies addressed potential beneficial effects of MSC on cardiovascular system in sepsis. In mice with endotoxemia, the cardiac function was impaired (reduced ejection fraction and fractional shortening) and application of bone marrow MSC prevented these functional changes [48]. Furthermore, MSC also reduced elevated levels of inflammatory mediators (IL-1*β*, IL-6, TNF-*α*, and IL-10) in both serum and myocardium. Expression of TLR-4, p65-nuclear factor-*κ*B, and phosphorylated p38 in endotoxemic myocardium was also reduced by MSC treatment. The data suggest that anti-inflammatory actions of MSC were able to reverse the detrimental effects of endotoxemia in the heart.

Similarly, in rats with endotoxemia, the treatment with MSC (both intraperitoneal and intravenous application) ameliorated the myocardial depression and reduced both serum and myocardial levels of inflammatory mediators (TNF-*α*, IL-1*β*, and IL-6). On the other hand, in contrast to mice, the serum and myocardial levels of an anti-inflammatory cytokine IL-10, which were increased by endotoxemia, were increased by MSC treatment even further [49].

The functional state, type, and gender of MSC may have significant impact on the potential therapeutic outcome of MSC treatment. Apoptotic adipose-derived MSC were reported to be superior to healthy adipose-derived MSC in treating rat sepsis induced by cecal ligation and puncture in terms of both reducing mortality and preserving organ function [50]. When effects of female and male MSC on myocardial function in rat endotoxemia were compared, female MSC treatment resulted in greater preservation of myocardial function [51]. The superior preservation of myocardial function with female MSC treatment was probably not related to anti-inflammatory effects of MSC since both serum and myocardial levels of cytokines were comparable between rats given MSC from male or female donors and also myocardial levels of phosphorylated p38 MAPK were similarly reduced by both male and female MSC. Endotoxemia was associated, however, with a decreased ratio of antiapoptotic and proapoptotic proteins suggesting a shift to increased myocardial apoptosis. Since this ratio was found to be significantly more increased in female MSC treatment than in male MSC treatment, the superior antiapoptotic effects of female MSC were suggested to contribute to better preservation of cardiac function with female MSC [51].

The mechanisms of beneficial cardiovascular effects of MSC will obviously require further attention and clarification. It has been documented that MSC, when infused systemically in septic animal models, home mainly to the lung and the liver but not the heart [52]. Therefore, the beneficial cardiac effects of MSC in sepsis are probably due to their systemic effects rather than local actions. Beside general anti-inflammatory and antiapoptotic effects, an interesting mechanism with potential therapeutic implications was reported recently: exosomal transfer of miR-223 [53]. In this study, MSC were shown to secrete miR-223-enriched exosomes, which were taken up by macrophages and cardiomyocytes. Consequently, the miR-223 targets were downregulated, leading to the inhibition of inflammatory response in macrophages and attenuation of cardiomyocyte death.

It should be emphasized that all the beneficial cardiovascular effects of MSC in sepsis were only described in small animal (mice, rats) models with limited clinical relevance so far. A thorough investigation of MSC effects in clinically relevant large animal models will be necessary before translation to clinical level.

## 6. Discussion

The immunomodulatory, anti-inflammatory, antiapoptotic, metabolomic, and antimicrobial effects of MSC undoubtedly form a legitimate biological basis for the scientific verification of their benefits and impact when used as adjuvant treatment not only in sepsis but also in a number of other critical conditions. Although this article is not an exhaustive systemic analysis or meta-analysis, it illustrates the comparable positive effects of MSC used in a relatively wide range of preclinical models of sepsis. These predominantly involve a positive effect on the mortality of septic animals and on the amelioration of AKI severity, as one of the most frequent end-organ dysfunction in sepsis. Does this mean that there is sufficient scientific basis for translating this research evidence into clinical practice, that is, for launching clinical trials? Certainly not!

The excitement sparked off by the potential therapeutic applications of MSC in medicine is understandable. However, there are a number of important reasons supporting a more reserved position on this issue. If we are to abide by the principles of scientific evidence, we must first and foremost unequivocally demonstrate not only efficacy but also safety. The clinical application of MSC that we are currently witnessing in the field of orthopaedics or neurology and which is based on minimal evidence of benefit and safety represents a path that critical care medicine should avoid. Its history has repeatedly shown that, thus far, no new therapeutic approach that was successfully tested in preclinical models was found to be effective in clinical testing (or on the contrary was shown to actually have a negative effect) [54]. There are specific reasons why generally homogenous and encouraging results attained by current preclinical testing cannot be considered as sufficient arguments for launching clinical trials. Firstly, there is a high risk that the effect of MSC is overstated given that a number of studies with negative results have not been published. For example, it has been documented that in the field involving research of stroke, 1 out of 6 studies was not published [55]. Secondly, all studies published thus far have exclusively involved rodents, mainly mice. The marked difference in the immune-inflammatory response to insults between rodents and humans is well documented [56]. Moreover, all preclinical studies discussed above have been carried out on inbred, young, healthy animals with a uniform genetic makeup and thus expressing none of the comorbidities. These models, however, might not reliably mirror the typical septic patient. Both aging and comorbidities not only increase the susceptibility to sepsis and sepsis-driven multiorgan dysfunction but may also influence the immune-inflammatory phenotype and, thus, the efficacy of MSC. Ideally, preclinical studies should use animal population of advanced age and with various comorbidities, such as diabetes mellitus, hypertension, atherosclerosis, and chronic kidney disease. Thirdly, in a number of experiments, the model of sepsis does not correspond to current requirements for clinically relevant biomodels (e.g., induction by endotoxins, rapidly lethal models, and absence of standard supportive treatment of sepsis, i.e., fluid resuscitation, vasopressors, antibiotics, and artificial lung ventilation). Long-term (days) realistic models with true focus on infection allowing the animals to develop full spectrum of typical hemodynamic, metabolic, immune-inflammatory, and tissue morphological responses rather than short-term (hours), rapidly lethal models should be used in examining both safety and efficacy of MSC in sepsis and multiorgan dysfunction. Fourthly, the source, dose, and timing of MSC are highly heterogeneous and remain open for discussion. Again, in many experiments, MSC were administered either concurrently or shortly after sepsis induction, a fact that significantly limits the translational potential of these results. Fifthly, and the last, the long-term consequences of treatment involving MSC are not known (e.g., the risk of developing malignancies, autoimmune states). Taken together, all the abovementioned facts should be appreciated and precisely elucidated before the results of any experimental work obtained from a single species/model are applied to other animals or even humans.

## 7. Conclusion

In summary, we may conclude that the encouraging results of experiments with MSC in sepsis represent sufficient background for further scientific analysis in the form of properly randomised trials using clinically relevant animal models. Only such models may confirm both the internal (methodological quality, bias risk) and external (i.e., generalisation) validity of experiments conducted to date. The decision to move from experiments to clinical studies should always be preceded by robust preclinical evaluation extending from small animal models to highly complex large models, ideally in the form of multicentre projects in several world-renowned experimental laboratories. One such monocentric project is currently under way at the authors' institution (project AZV, 15-32801A) and results maybe expected next year. It is thus even more surprising that two clinical studies of MSC in sepsis have already been registered (NCT02883803, Effects of Administration of Mesenchymal Stem Cells on Organ Failure During the Septic Shock (CSM choc); NCT02421484 Cellular Immunotherapy for Septic Shock: A Phase I Trial).

## Figures and Tables

**Table 1:** Preclinical models of sepsis and role of MSC.

Authors/year	Sepsis model	MSC type/combination	Biological effect	Clinical effect	Source ref.
Asano et al. (2015)	TSS SEA + LPS mouse model	A-MSC (1 × 10^6^)	↓ INF-*γ*, TNF-*α*, IL-6, IL-2 = Treg, IL-10	↓ 40 h mortality	[25]
Kim et al. (2014)	TSS SEB mouse model	hMSC, mMSC (2 × 10^5^)	↓ TNF-*α*, IL-2, IL-6	= mortality No difference between hMSC and mMSC	[26]
Ou et al. (2016)	LPS mouse model	A-MSC, BM-MSC	↓ IL-8 (A-MSC) ↓ proinflammatory cytokines (both types)	↓ mortality	[27]
Pedrazza et al. (2014)	*E. coli-*induced peritonitis	A-MSC (1 × 10^6^)	↓ IL-6, MCP-1 ↓ AST, ALT ↓ splenocyte apoptosis	↓ 26 h mortality	[28]
Chao et al. (2014)	CLP-polymicrobial mouse model	BM-MSC UC-MSC (5 × 10^6^)	↓ IL-6 and TNF-*α* ↑ CD3^+^CD4^+^CD25^+^ Treg	↓ 7- and 14-day mortality	[29]
Alcayaga-Miranda et al. (2015)	CLP-polymicrobial mouse model	Men-MSC, A-MSC, BM-MSC (2 × 10^6^)/enrofloxacin	*In vitro*: ↑ inhibition of bacterial growth (Men-MSC) No difference—Men-MSC versus A-MSC/BM-MSC in the dynamics of cytokines	Men-MSC + ATB ↓ 96 h mortality	[30]
Wang et al. (2015)	CLP-polymicrobial mouse model	D-MSC (2 × 10^6^)	↓ IL-1, IL-6 ↑ IL-4, IL-5 IL-10 without significant changes *In vitro*: inhibition of macrophage apoptosis, increased migration intensity	↓ 10-day mortality	[31]
Liu et al. (2016)	CLP-polymicrobial mouse model	Unspecified (1 × 10^6^)	↓ NK ↓ TNF-*α*, IL-6, INF-*γ* ↑ IL-10	↓ 72 h mortality	[35]
Sepúlveda et al. (2014)	LPS mouse model	BM-MSC (1 × 10^5^)	*In vitro*: no difference between senescent versus immortalised *In vivo*: no reduction of proinflammatory cytokine levels in senescent cells	Immortalised MSC: ↓ 24, 48, 72, 96, 120, and 144 h mortality versus senescent type	[36]
Wu et al. (2016)	CLP-polymicrobial sepsis	UC-MSC	↓ TNF-*α*, MCP-1, IL1, 6 ↑ IL-10 ↓ mRNA MyD88 ↓ phosphorylation NF-*κ*B	↓ 6 h mortality	[37]

TSS = toxic shock syndrome; SEA, B = staphylococcal enterotoxin A, B; LPS = lipopolysaccharide; CLP = cecal ligation and puncture; A-MSC = adipose tissue-derived MSC; hMSC = human MSC; mMSC = mouse MSC; Men-MSC = menstrual-derived MSC; BM-MSC = bone marrow-derived MSC; D-MSC = dermal MSC; UC-MSC = umbilical cord MSC.

**Table 2:** Preclinical models of S-AKI and the effect of MSC.

Team/year	Animal model	Type of MSC/combination	Effect of MSC	Ref.
Luo et al. (2014)	CLP-polymicrobial mouse model	Unspecified MSC (1 × 10^6^)	↓ urea, creatinine ↓ IL-17, CXC, CCL ↓ ATN score	[41]
Cóndor et al. (2016)	CLP-polymicrobial rat	WJ-MSC (1 × 10^6^)	↑ glomerular filtration (inulin clearance) ↓ apoptosis intensity in the renal parenchyma ↓ kidney infiltration by immunocompetent cells	[42]
Sung et al. (2013)	CLP-polymicrobial mouse model	Apoptotic MSC (1.2 × 10^6^)	↓ TNF-*α* ↓ serum creatinine	[43]
Tsoyi et al. (2016)	CLP-polymicrobial mouse (BALB/C)	MSC (2.5–5 × 10^5^) CO preconditioning	↓ incidence of AKI	[44]
Sung et al. (2016)	CLP-polymicrobial mouse model l	A-MSC (5 × 10^5^)/ciprofloxacin (3 mg/kg/5 days)	↓ expression of proinflammatory cytokines in the kidney	[45]
Chen et al. (2014)	CLP-polymicrobial rat	A-MSC (1.2 × 10^6^)/melatonin (20 mg/kg)	↓ levels of proinflammatory cytokines ↓ expression of NF-*κ*B in the kidney	[46]

WJ-MSC = MSC derived from Wharton's jelly; AKI = acute kidney injury; CO = carbon monoxide.
